# Internet-Based Direct-to-Consumer Genetic Testing: A Systematic Review

**DOI:** 10.2196/jmir.4378

**Published:** 2015-12-14

**Authors:** Loredana Covolo, Sara Rubinelli, Elisabetta Ceretti, Umberto Gelatti

**Affiliations:** ^1^ Unit of Hygiene, Epidemiology and Public Health Department of Medical and Surgical Specialties, Radiological Sciences and Public Health University of Brescia, Italy Brescia Italy; ^2^ Department of Health Sciences and Health Policy University of Lucerne and Swiss Paraplegic Research Lucerne/Nottwil Switzerland

**Keywords:** genetic testing, direct-to-consumer, Internet, online market, systematic review

## Abstract

**Background:**

Direct-to-consumer genetic tests (DTC-GT) are easily purchased through the Internet, independent of a physician referral or approval for testing, allowing the retrieval of genetic information outside the clinical context. There is a broad debate about the testing validity, their impact on individuals, and what people know and perceive about them.

**Objective:**

The aim of this review was to collect evidence on DTC-GT from a comprehensive perspective that unravels the complexity of the phenomenon.

**Methods:**

A systematic search was carried out through PubMed, Web of Knowledge, and Embase, in addition to Google Scholar according to the Preferred Reporting Items for Systematic Reviews and Meta-Analyses (PRISMA) checklist with the key term “Direct-to-consumer genetic test.”

**Results:**

In the final sample, 118 articles were identified. Articles were summarized in five categories according to their focus on (1) knowledge of, attitude toward use of, and perception of DTC-GT (n=37), (2) the impact of genetic risk information on users (n=37), (3) the opinion of health professionals (n=20), (4) the content of websites selling DTC-GT (n=16), and (5) the scientific evidence and clinical utility of the tests (n=14). Most of the articles analyzed the attitude, knowledge, and perception of DTC-GT, highlighting an interest in using DTC-GT, along with the need for a health care professional to help interpret the results. The articles investigating the content analysis of the websites selling these tests are in agreement that the information provided by the companies about genetic testing is not completely comprehensive for the consumer. Given that risk information can modify consumers’ health behavior, there are surprisingly few studies carried out on actual consumers and they do not confirm the overall concerns on the possible impact of DTC-GT. Data from studies that investigate the quality of the tests offered confirm that they are not informative, have little predictive power, and do not measure genetic risk appropriately.

**Conclusions:**

The impact of DTC-GT on consumers’ health perceptions and behaviors is an emerging concern. However, negative effects on consumers or health benefits have yet to be observed. Nevertheless, since the online market of DTC-GT is expected to grow, it is important to remain aware of a possible impact.

## Introduction

“There’s no gene for fate.” This is a quote from the movie “Gattaca,” a 1997 American science fiction film set in a future when one’s life is determined by genetic engineering rather than education or experience [1]. This theme expressed concern about the negative effects of a genetic determinism foreseen in a distant future. However, only a few years later, advertisements such as “Your future health is in your genes,” [2] “Your DNA, your personal health,” [3] or “Diet and exercise matched to your genes” [4] started to appear on websites of commercial companies offering direct-to-consumer genetic testing (DTC-GT). There are even companies offering tests to find genetic compatibility with a partner, presented as a key to successful and long-lasting romantic relationships [5]. One can imagine this scenario triggering a genetic determinism in potential consumers, mainly because there is no involvement from health professionals. The paradox is that, despite the fact that predictive genetic tests are already on the market, the majority of such tests lack scientific evidence and a proven clinical utility [6,7].

Over the past decade, the phenomenon of DTC-GT has generated a huge debate among physicians, bioethicists, and government bodies [8-12], and many recommendations are available [13-15]. In November 2013, the US Food and Drug Administration (FDA) ordered 23andMe, a provider of DTC genomic services, to stop marketing health-related genetic tests due to the risk that false results could cause consumers to undergo unnecessary health procedures [16]. However, there are currently other online companies offering this kind of service [2-4,17].

The current evidence on the risks of DTC-GT is uncertain. To our knowledge, there are three main systematic reviews on specific aspects related to DTC-GT [15,18,19]. These reviews, carried out by the same group of authors, separately explored the current position statements and recommendations on the use of DTC-GT [15], along with the views and experiences of consumers [18] and health professionals [19]. Analysis of documents produced by professional or public organizations [15] has caused great concern about potential harms for consumers who might undergo DTC-GT. Considering the difficulty in creating international standards that regulate the online market, the authors underlined the need to promote an agreement on a code of practice based on specific recommendations that include appropriate education for health professionals, as well as the guarantee of appropriate information to consumers. But there are mixed views on the actual risks of DTC-GT. With evidence that DTC-GT might actually increase the demand for consultation and related screening or diagnostic testing, some health professionals rated GT as clinically useful and a valuable opportunity for early screening [19].

There are two additional recent reviews on DTC-GT [20,21] that explore this topic in general and conclude that, from the consumer’s experience, there does not seem to be enough evidence to qualify the risks of these tests. Yet, these two reviews were not based on a systematic approach.

The objective of our review is to merge evidence on DTC-GT from a more comprehensive perspective than the studies mentioned above. In addition to identifying further literature on the value of DTC-GT from the point of view of consumers and health professionals, this review also considers the scientific evidence and clinical utility of this type of testing and the way DTC-GT is marketed from a health communications perspective. The analysis of these last two aspects are essential to offering a multifaceted framework for understanding the complexity of DTC-GT as a phenomenon and informing directions for future research and policy making in the field.

## Methods

The systematic review was performed according to Preferred Reporting Items for Systematic Reviews and Meta-Analyses (PRISMA) guidelines [22] (see Multimedia Appendix 1).

### Information Sources

The literature search covered the period up to October 2014. The search was performed using electronic databases (PubMed, Web of Science, and Embase) and the Google search engine tool, Google Scholar. On Google Scholar, we investigated all the results obtained by the databases, but considered only the first 500 results because the number of relevant articles declined substantially after the first 300 results and because this search engine displays results by relevance using a link analysis system or algorithms [23].

### Search Strategies

We used “Direct-to-consumer genetic test” as the key term for each database and for Google Scholar. We scanned the reference lists for relevant articles up to the second level, and we considered the “related articles” of relevant ones in the PubMed database or Google Scholar when the paper was not present in PubMed.

### Study Selection and Eligibility Criteria

We included all articles relevant to the subject of the research where the key term was anywhere in the text of the paper, written in English, with the abstract and full text available. We included only scientific articles, excluding popular articles published in daily newspapers or weekly and monthly magazines. Papers included articles associated with health-related genetic tests available online and offered direct-to-consumer. We selected only the articles reporting original data, excluding those with speculative discussion about the problem or citing data from other studies (ie, editorials, letters, comments, articles about regulation issues, and reviews).

Two investigators read the papers (LC and UG) and independently assessed the potential relevance of all publications, identified during the database search, based on the information provided in the titles and abstracts. Disagreement was resolved by consensus.

**Figure 1:**
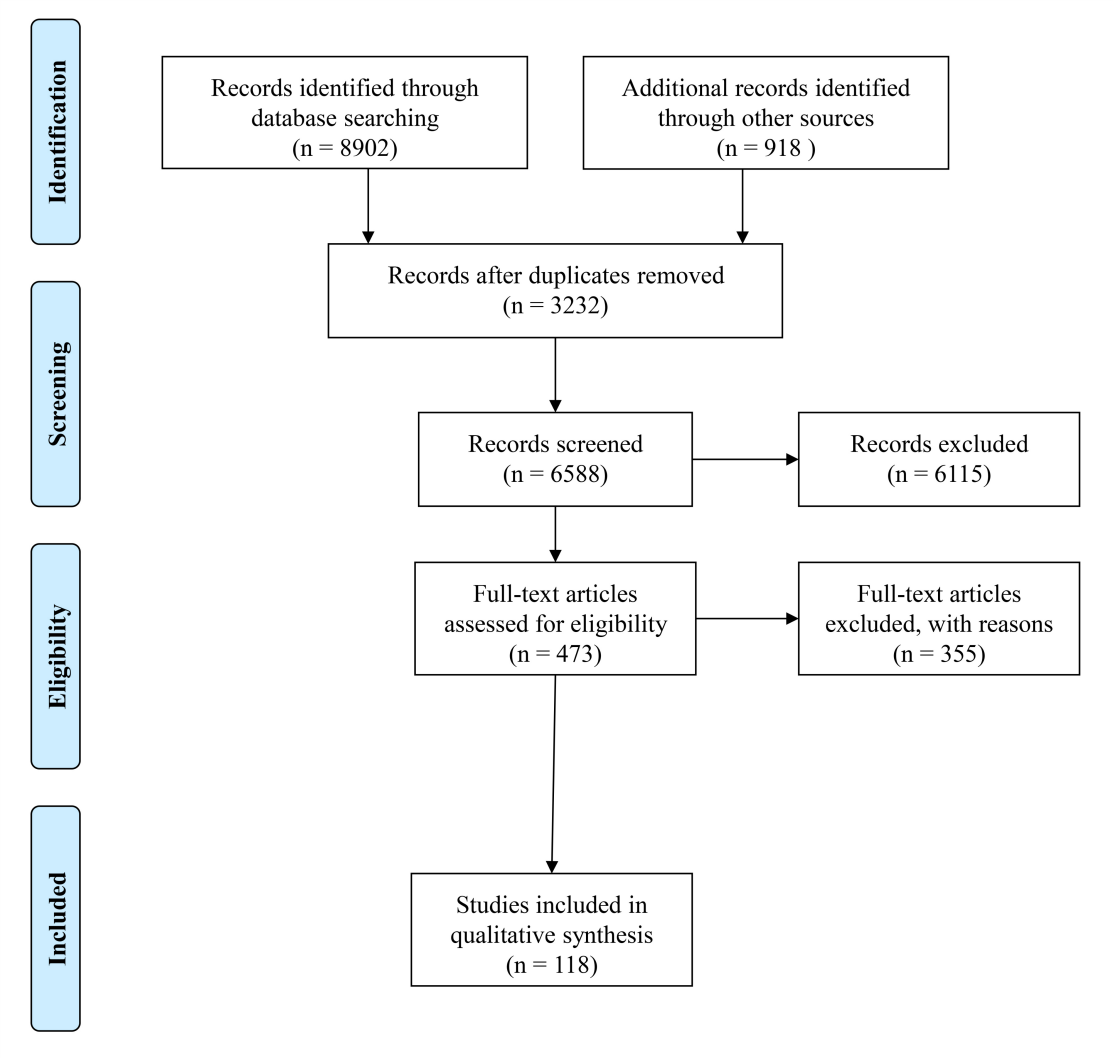
Flow diagram describing the study selection.

After screening by titles and abstracts, the first author critically reviewed the full texts of the remaining articles and extracted the information required to perform the review. The methodological quality of each study was assessed by 2 authors (LC and EC) using the Kmet tool for evaluating quantitative and qualitative research [24]. A score of between 0 and 1 was assigned to each paper based on a series of questions related to the type of study. Case studies and descriptive reports (a total of 38 papers) were excluded from the evaluation. Disagreements were resolved through discussion among the authors until consensus was reached. As shown in Figure 1, we identified 118 articles that fit the inclusion criteria.

## Results

**Figure 2:**
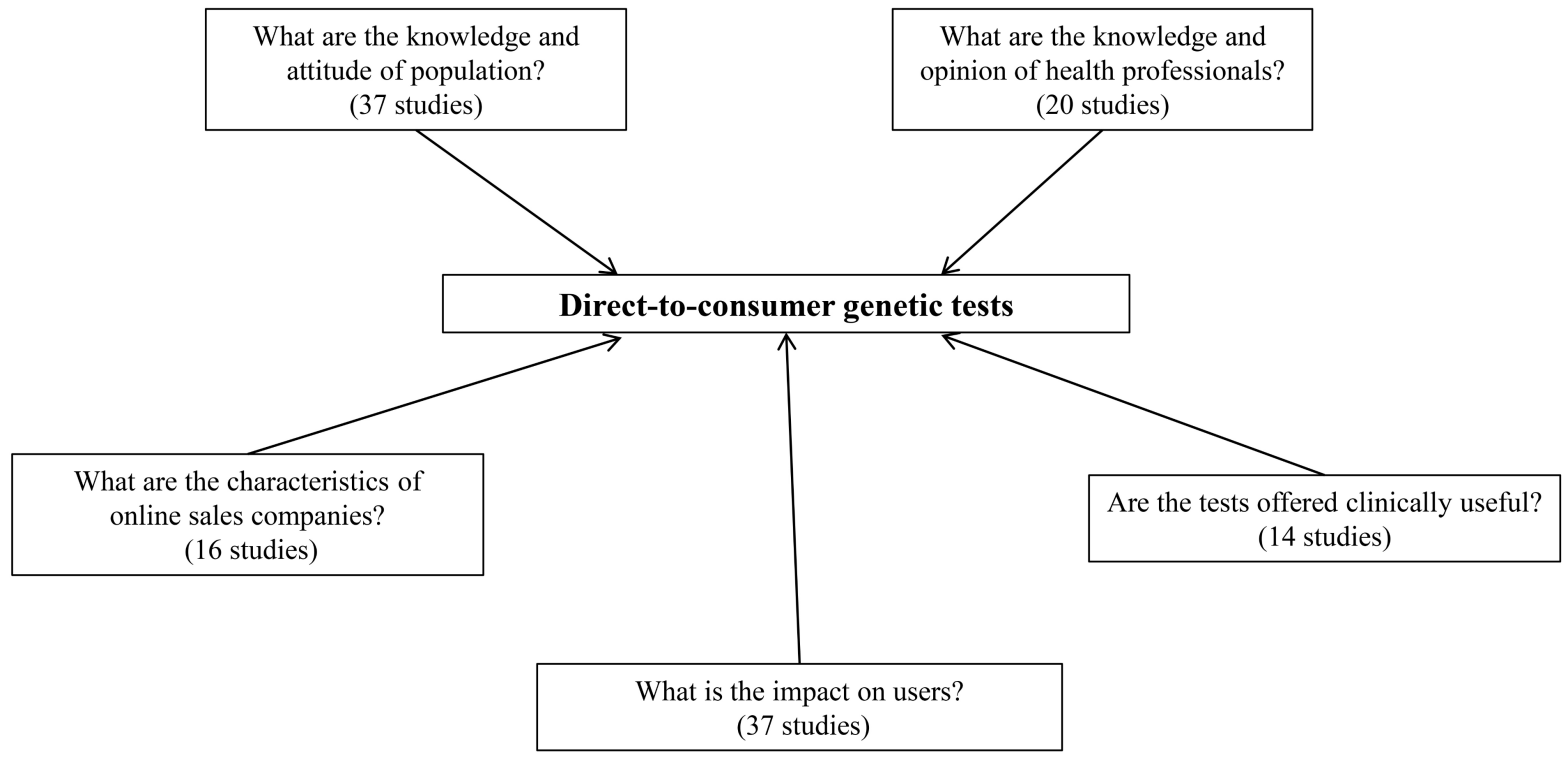
Research categories.

Of the 473 relevant articles selected, we included 118 studies with original data (24.9%): 95 quantitative studies, 15 qualitative studies, and eight case studies. These articles have been divided in five categories as shown in Figure 2: (1) knowledge and attitude/perceptions to DTC-GT, (2) health professionals’ opinions about DTC-GT, (3) characteristics of online companies selling GT, (4) DTC-GT’s impact on users, and (5) evidence of clinical utility and validity. Some articles with original data covered more than one of these subjects and were consequently allocated to more than one group. For studies investigating DTC-GT’s impact on users, we included studies investigating both hypothetical situations (n=20) and actual situations (n=17).

### Research Categories

#### Public’s Knowledge of and Attitude Toward Direct-to-Consumer Genetic Testing

We selected a total of 37 articles, of which four are qualitative studies, investigating the public’s knowledge of and interest in DTC-GT [25-28] (see Table 1 in Multimedia Appendix 2). Of the 37 studies, 25 (68%) were carried out in the United States [25-50] with 41% (15/37) registering more than 1000 subjects [30-33,37,40,42,43,46,51-56]. The response rate was more than 60% in over half of the studies (16/30, 53%) where such data were available [30,32,33,35,36,38,40,45,47,50,52,56-60]. In general, the age range of participants was quite wide with a mean age ranging from about 25-50 years. The education level was high (college degree or more) for the majority of participants in all studies.

Overall, the level of awareness of DTC-GT was low, ranging from 8% [33] to around 50% [37,43,45,57]. In two studies with a large number of subjects (more than 4000) and a response rate of more than 60%, there was a very low level of knowledge of DTC-GTs, specifically 13% [59] and 14% [32].

A total of 78% of subjects recruited in the study by Gollust et al [34] were aware of personal genomics, but only 15% visited a DTC-GT website. A large portion of women heard about GTs (73%) in the study by Perez et al [47]. However, the sample was small (84 women) and characterized by women at high risk for breast cancer who may have been more aware of this subject.

In 4 studies [25,30,35,54], the participants expressed great interest in GTs. In fact, 82% of subjects recruited at the Scripps Transitional Science Institute reported that they would want to know their disease risk [30]. However, as highlighted by the authors, the sample was not representative of the general public because it was largely made up of Institute employees and a number of technology and biotechnology company employees. A large number of women indicated definite interest in GT (77%) in the study by Graves et al [35], but they were women at moderate to high risk for breast cancer. Similarly, the interest in having GT to determine susceptibility to major depression was higher in participants affected by a depressive disease than those unaffected (71% vs 64%), although not statistically significant [25]. In another seven studies [27,42,43,55,57,59,60], a moderate interest in GT (from 50-60%) was found. It should be noted that 48% of respondents in the Cherkas et al [59] study were interested in GT if the test was free of charge. Similarly, 37% of a sample of Canadian adults stated that they would pay nothing for GTs even if related to a manageable condition [53]. Only 5% were potentially interested at the current price (£250). In several studies, fewer than 40% of participants expressed favorable attitudes to GT [36,37,47,48,52,56,58].

The interest in GT seemed to increase only when the information received was positive [49], when people felt they would regret not taking the test [54] or, in the case of parents, when they could learn about their child’s decreased risks [50]. Survey respondents who perceived greater threat from disease had significantly greater behavioral intentions to talk to their doctor and search for more information about the test, even if it did not affect their plans to take the test [61]. Additionally, when people were informed about the risks of DTC testing, they became less interested in getting GT [36]. At the same time, it was found that conscientiousness about the risk of GT, and not neuroticism, led people to seek online information about DTC-GT [46]. Web-based genomic information presented using evidence-based communications made patients more favorable to this type of testing [38].

The importance of having information about GT was also supported by the need to refer to a physician to interpret the test results [34,41,43,56]. Nearly half (46%) of women recruited in the Perez et al study [47] strongly agreed that it is more appropriate for companies to target doctors to identify women who may be at risk for carrying the breast cancer gene than target all women through different types of media. Respondents of a large Australian survey [51] were not comfortable with companies offering DTC-GT and were unlikely to order the test because it was perceived to be less regulated and accurate compared with a test provided by a conventional medical practitioner. Concerns about poor regulation of DTC-GT companies and violation of privacy emerged from media coverage of DTC-GT [26].

Among the reasons for favorable attitudes to GT were curiosity [34,43,59] and interest in monitoring and improving health [28,34,57,59]. University students in Switzerland reported the contribution to scientific research as their main reason for undergoing testing [55]. The availability of treatment was a factor that motivated the respondents of a Canadian sample population (61%), whereas curiosity had only a modest impact on willingness to pay for GT. Younger respondents were more likely to cite curiosity as a reason for testing [53].

#### Impact of Direct-to-Consumer Genetic Testing on Users

We retrieved a total of 29 articles dealing with the consequence of undergoing a DTC-GT, of which nine were qualitative studies [62-70] (see Table 2 in Multimedia Appendix 2). A qualitative analysis was also included in the study of Vayena et al [71]. Furthermore, there were eight case studies [72-79]. As much as 68% (25/37) of the studies, including case studies, were carried out in the United States [32,43,63-66,70,73,75-79, 81-93]. Excluding case studies, nine studies investigated the DTC-GT experience with people who actually purchased the test [32,43,62,67-69,90,91,94]. In the other studies, participants were investigated only as potential consumers. Overall, the main goal was to evaluate psychological reactions, behavioral effects, and perception risk.

In 41% of studies (12/29) [32,43,62-68,70,71,90], the sample size was very low (fewer than 100 subjects). There are some exceptions: Kaufman et al [91] recruited 1048 subjects (but with a response rate of only 33%) and Su et al [69]. Also, six studies [81-83,85,86,93] referred to the same large sample coming from the longitudinal cohort study of 3639 adults recruited from Scripps Health employees, employee family members, and Scripps Health patients who purchased the GT at a discounted rate [30].

Regarding the impact of GT results on health behavior, a large proportion of participants expressed the intention to modify lifestyle (eg, diet, exercise), both among actual customers [43] and hypothetical ones [55,63,80], and a modest change in health behavior was observed, particularly among people who purchased a DTC-GT [67,91,94]. In the study by Francke et al [90], 11 out of 16 women received information about being positive for the breast cancer type 1 susceptibility gene mutation (BRCA) from the DTC company. Although the number is modest, three of them had risk-reducing oophorectomy and four planned to. One had a mastectomy and three planned to. Five declared they went to have breast exams and breast imaging after getting their results.

No impact on potential user behavior was evidenced at the 3-month follow-up [93] or after a moderately longer period of observation (1 year) [65,82,92]. No influence on DNA-based dietary advice in personalized nutrition perception was observed in a randomized control trial after 1 year of follow-up [95].

Slightly more than half of the people who used a DTC-GT discussed their results with a physician [43]. Similarly, 60% of 23andMe customers, who showed as mutation-positive, reported sharing their results with their physician. Only 26% of the mutation-negative customers shared their information with their physician [90]. Increased physician utilization was found among people who underwent DTC pharmacogenetic tests [83].

In general, the number of people who reported sharing test results with a physician is quite low (<30%), both among actual users [32,67,91] and experimental ones [66,70,71,86], even though most participants stated that they would (or might) disclose to physicians when asked in the study by Wasson et al [66]. After the 1-year follow-up, no changes were found in the overall use of health care by those receiving personalized GT results compared to those who were not tested [92].

Generally, the proportion of people worrying about their tests results was also quite low. Fewer than 30% of DTC-GT customers declared a change in health anxiety [94] or felt anxious even if mutation-positive [90]. Furthermore, there was no significant difference between baseline and follow-up anxiety symptoms at 3 months [81,89] and 1 year [82,83,87] after receiving test results. Bloss et al [81,82] found that greater perceived seriousness and diminished perceived control over a disease were associated with test-related distress and higher, but not clinically significant, levels of anxiety [93]. In addition, people who shared their test results with health care providers were significantly less worried about being tested compared to non-sharers (45% vs 53%, *P*=.01), but only a small percentage (around 10%) were worried about learning of disease risk in both groups. Nevertheless, great value was attributed to risk information in 78% of sharers and 69% of non-sharers (*P*<.01) [86].

Several studies showed no concern by all or a majority of participants [55,63,65,71]. Concerns seemed to relate to the type of results. For example, a significant increase in negative effect was shown among individuals who learned that they were susceptible to alcoholism [84]. However, the intentions for alcohol consumption in the near future were not affected. In a study by Gordon et al [70], 88% of participants reported feeling reassured by these test results: indeed, they were encouraged by learning of their negative test results and their low-risk factor. Similar conclusions were found by Harris et al [62] who analyzed stories told by DTC-GT users. These participants even felt a sense of indifference toward the test results. On the contrary, almost half of people who knew about a cancer risk and 81% of people who learned about myocardial infarction risk through a DTC-GT were worried about these diseases. After 1 year, there were no differences in being worried compared to people who had not been tested [88].

People who did not interpret test results as deterministic on health outcomes, or declared they understood the results, were generally not worried about them [63,70,87,89]. People who were tested for four conditions perceived a higher risk than those who were not tested; a large portion of them even expressed concern about their disease risk. This difference was, however, not significant 1 year after receiving test results [88]. Only 10% of people interviewed in the study by Vayena [71] reported a serious impact on their health perception, 55% stated some impact, and 35% affirmed no impact, while less than half of these respondents reported having no concerns at all about DTC testing. It should be noted that their primary reported concerns regarded privacy issues.

In another context, it was interesting to find that many people signed a petition to support unrestricted access to DTC-GT stating also the health care professional and government should not be placed as intermediaries when purchasing DTC-GT [69]. The perception of having understood the test results was the main reason for not utilizing the counseling service [82]. However, the evidence showed that the delivery of personal genomic risk through a trained health professional resulted in significantly higher comprehension compared to online delivery [87].

An incorrect interpretation risk was found among people who underwent DTC-GT [91], and the majority of DTC consumers interviewed by McGuire et al [43] considered information obtained from DTC-GT to be a diagnosis of a medical condition. This evidence contrasts with other studies that revealed that many people were aware of the low predictive value of DTC-GT [67] or the fact that they report an average risk of disease [94]. The main reason for purchasing the GTs related to health, as well as a general curiosity about genetic make-up [43,62].

#### Case Studies

We retrieved seven studies reporting on patients who purchased a DTC-GT and one analyzing information from two reports from a DTC-GT company [73]. Except for two case studies in which DTC-GT were considered useful [77,78], all other case studies underlined the importance of correctly understanding and interpreting the results in order to avoid adverse psychological consequences [73,74,76], unnecessary preventive measures [79], or the possibility of giving the genetic profile a deterministic role [72]. This is particularly important when people learn about their susceptibility to Alzheimer’s disease for which proven preventive strategies are still lacking. On the basis of 2 subjects who tested positive for Alzheimer’s, the need to improve strategies for informed decision making was discussed. For example, DTC-GT could provide a more detailed consent form and promote a mandatory pre-test conversation with a genetic counselor. This is highly relevant for DTC-GT as the health information provided by DTC companies seems to be influenced by commercial loyalties and can therefore be potentially misleading [74].

Learning about a genetic predisposition to curable diseases may be beneficial, as was the case for a woman who learned from her DTC-GT that she was at high risk to develop breast cancer. She felt empowered by prevention, although it was genetic counseling that contributed to her facing and limiting her initial anxiety [75]. The support of a health professional is also crucial when considering the risk of misinterpreting the test results [73]. Another study reported the case of a 52-year-old man whose test results implied that his obesity was genetically predetermined and inevitable, but after appropriate lifestyle modification he lost 32 kg, indicating the importance of environmental factors [72]. In this context, Corpas [74] highlighted the need to have psychological support in sharing results with family, an aspect that is not emphasized in the DTC-GT process.

### Health Professionals’ Point of View on Direct-to-Consumer Genetic Testing

A total of 20 articles explored health professionals’ opinions of DTC-GT as reported in Table 3 in Multimedia Appendix 2. Two of these are qualitative studies [96,97]. Out of 20 studies, 14 were mainly conducted in the United States (70%) [32,41,97-108]. Half of the surveys were implemented online [32,41,98-101,107-110].

Four studies recruited more than 1000 subjects [32,102,108,111], and five studies recruited more than 300 subjects [52,98,101,105,107]. Almost half of the studies (44%) had a response rate of around 40% [100-102,104,109,110,112] and other 6 studies (38%) had a response rate less than 20% [41,99, 103,105-107,111].

The awareness of DTC-GT among physicians was high (around 90%) in three studies [41,100,110]. In other surveys of physicians not specializing in genetics, fewer than 55% of respondents were aware of DTC-GT [102,105,111,112]. In general, the percentage of physicians who have discussed GT results with a patient or have ordered a GT for a patient was quite low. Sixteen percent of physicians recruited by Bernhardt et al [98] ordered one test a week or more, and only 7% reported having seen a DTC genetic risk assessment report. In five surveys, fewer than 19% of physicians, both specializing in genetics and not, reported having patients request genetic consultations [32,99,105,107,108]. Forty-four percent of clinical geneticists from different European countries had been contacted by at least one patient regarding DTC-GT services after purchasing the test [110], and 46% of genetic counselors from the United States had worked with patients who had initiated a discussion of DTC-GTs: however, only 15% of the latter had suggested them to their patients [101]. Forty-two percent of primary care physicians enrolled in an online survey in the United States had ordered a GT for a patient, and one third had ordered them for themselves [100]. Only 0.5% of general practitioners and 1% of clinical geneticists from Japan ordered DTC-GT [111]. A large percentage of neurologists (74%) and the 14% of psychiatrists recruited from the American Medical Association ordered a GT for a patient [107].

Another interesting topic is how confident physicians are in interpreting GT results. In the study by Bernhardt et al [98], 16% of primary care physicians declared themselves to be “very confident,” along with 15% of family physicians in the study by Powell et al [106] and only 7% of physicians specialized in genetics from the study by Brett et al [109]. In a study on nutrigenomics, health professionals reported a lack of competency to provide information on nutritional genomics. Inability to support a patient in managing genetic risk information also emerged from interviews with 18 clinicians providing genomic risk assessment services to their patients [97]. The study by Salm et al [107] reported the need to have more training in interpreting GT results; although in the context of predictive genomic testing, the United States has promising training programs for genetic counselors [103].

Health professionals’ opinions on the clinical utility of DTC-GT were contrasting, and the percentage of those who were in favor of these services was different among the studies. Giovanni et al [99] found that 52% of health care providers described the genetic test as clinically useful. The majority of respondents (86%) mentioned usefulness in the context of breast cancer susceptibility, in agreement with findings from Mainous et al [102]. The latter also found that 30% of participants perceived GT utility in detecting Alzheimer’s disease and 25% of participants for heart disease or diabetes. Half of genetic counselors recruited by Hock et al [101] said that GT should be limited to a clinical setting, 23% of the sample was neutral, and 27% disagreed. Furthermore, 56% of the sample considered a DTC-GT acceptable only with the provision of genetic counseling, 31% were neutral, and 13% disagreed. In the study by Bernhardt et al [98], physicians thought that genetic tests would be helpful in managing patients; in particular, 70% felt it would be useful with pharmacogenomics and 40% with disease risk assessment. However, only one third of physicians in both cases would order such testing for a patient. About 47% stated that genetic testing would be helpful for patients, motivating them to adopt healthy behaviors. Also, the clinicians interviewed in a recent study [97] were enthusiastic about the potential of GTs to enhance the personalized, preventive, and wellness orientations of their clinical practices.

In a study conducted in Australia [109], the majority of genetic health professionals did not consider DTC-GT useful for individuals who want anonymous testing (54%), are driven by curiosity (54%), or are geographically isolated (60%). Forty-three percent of physicians in the Powell et al [105] study considered DTC-GT clinically useful. In a Greek study by Mai et al [52], only 13% of medical practitioners were in favor of DTC-GT. Similarly, 86% of clinical geneticists recruited from 28 European countries [110] considered it unacceptable to provide a predictive test without face-to-face medical supervision, and all respondents expressed the unacceptability of offering DTC-GT for conditions neither treatable nor preventable.

Ohata et al [111] carried out a survey on 1145 general practitioners and 294 clinical geneticists in Japan. Convenience scored highest in both groups as the reason behind users’ ordering DTC-GT, and general practitioners rated the benefits of DTC-GT higher than clinical geneticists (score 2.54 vs 1.96 on a scale 1-4, 1=disagree). Among the risks, the concern for understanding results scored highest in both groups (score >3). Furthermore, reliability of results and provision of information/counseling were a source of concern greater in clinical geneticists than general practitioners (score 3.13 and 3.78 vs 2.77 and 3.48 respectively).

In a study conducted in New Zealand [112], general practitioners who had not received training in genetics agreed that convenience was a benefit, more than those with training (72% vs 38%, *P*<.005). At the same time, misinterpreting results and inadequate delivery of information were perceived to be the greatest risks associated with DTC-GT by the majority of respondents (around 90%). In general, only 19% agreed that DTC-GT provides a useful service in the delivery of health care, and 26% agreed that results encourage patients to take responsibility for their health. Clinical validity of the test (25%) and counseling (20%) were the most selected aspects regarding advertising regulation of DTC-GT.

In another study [108] dealing with GT in children, genetic counselors appeared less prone to GT compared with non-genetic physicians.

There was one study exploring the knowledge of and attitude toward personal genomics on a small group of medical students enrolled in a human genetic course [104]. The percentage of students who thought that genotyping information would be useful to physicians and consumers decreased after the course (32% post-course vs 63% pre-course and 52% vs 84% respectively). The majority of students, both before and after the course, expressed concerns about reliability and utility of results. They agreed that tests needed interpretation (around 70%) and DTC companies had to provide genetic counseling (71% pre- and 80% post-course).

More than 80% of physicians recruited by Powell et al [105] expressed concerns about possible misinterpretation of test results and increased anxiety in patients. Almost half of physicians (neurobiologists and psychiatrists) surveyed by Salm et al [107] thought that GT could cause psychological harm to their patients and they could be exposed to possible insurance discrimination. This was further confirmed in the study by Bernhardt et al [98].

Uncertainty about clinical utility concerned the majority of primary care physicians (around 60%) in the study of Haga et al [100], with a recommendation for health care professionals to act as intermediaries also when discussing DTC nutrigenomic tests [96].

### Content of Websites Offering Direct-to-Consumer Genetic Testing

A total of 16 articles were identified regarding issues and marketing strategies related to the type of information provided by the DTC-GT websites (see Table 4 in Multimedia Appendix 2). The number of websites analyzed ranged from three [113] to 38 [114].

Goddard et al [115] found 27 health-related DTC-GT distributor websites and evaluated those that sold tests for thrombosis risk. Liu et al [116] analyzed 46 websites, but only 20 of them allowed consumers to order directly from the company. Sterling et al [117] identified 64 organizations hosting websites promoting nutrigenomic services, but only 29 offered or promoted at-home testing.

Borry et al [118] and Howard et al [119] investigated online companies focusing mainly on their policies in regard to GT for minors. The former analyzed 29 companies obtained from a list published by the Genetics and Policy Center, and the latter sent a questionnaire to 37 DTC-GT companies. Both studies emphasized a lack of exhaustive information on the privacy policy regarding minors, which is a deviation from the professional guidelines on this issue. Also, in a recent systematic Internet search for DTC genomic services, limited information on privacy policies was found [120]. This evidence contrasts with other studies that found the majority of sites selected provided this information [115-117,121,122].

Most studies assessed the quality of information provided by online GT companies through a content analysis of websites, with a focus on the provision of genetic counseling, suggestion for a physician’s consultation, and the description of risks, benefits, and limitations of GTs.

In relation to online genetic counseling, Geransar et al [123] showed that of the 24 online companies studied, 75% recommended and arranged for counseling services. However, only one-third of the companies directly provided counseling services and just one of them provided a face-to-face format. Half of the websites analyzed by Covolo et al [124] provided this service pre- and/or post-test, with 20% offering this service for an extra fee. In other cases, fewer than 39% of online companies provided genetic counseling [114,119,121,125,126]. Pre-test counseling was rarely offered in studies conducted by Hennen et al [114], Lachance et al [121], and Liu et al [116]. None of the 29 companies offering nutrigenomic services examined by Sterling et al [117] provided genetic counseling.

Additionally, except for the websites analyzed by some studies [115,123,124,127], very few companies suggested a physician’s consultation [114,117,119,121,125]. Sometimes the GT sale was accompanied by recommendations associated with disease prevention or health improvement (eg, nutritional supplements). This trend was found in the majority of websites (from 60-74%) investigated by Lewis et al [122] and Singleton et al [127]. Of 64 websites promoting nutrigenomic services identified by Sterling et al [117], 53% provided recommendations for dietary intake or supplementation.

Genetic discrimination, emotional consequences, risk of behavior changes, and confidentiality of test results are possible risks associated with GT. In general, all studies that searched for this information found that the risks were poorly cited, ranging from about 20% [115,116,122,128] to about 30-36% of the websites [117,124,125,127]. Of the company websites analyzed by Hennen et al [114], 47% provided information on consequences and actions to be taken in the case of a positive test result, and 37% in the case of a negative test result.

Clearly, the benefits of testing are described more than risks [115-117,124,127,128]. In particular, empowerment over one’s health was highlighted by several authors [113,116,124,128]. Almost all of the sites identified by Lachance et al [121] and Singleton et al [127] listed at least one benefit to consumers by undergoing testing. Three-quarters (76%) of websites analyzed by Lachance et al [121] highlighted the fact that test results can help inform consumers in making a health decision. In the second study, prevention of the onset of a disease was the most common benefit presented (96%). Interestingly, 52% of websites stressed the consumer’s ability to use the results to make informed decisions. The concept of patient empowerment also appeared in the Sterling et al [117] study. In fact, 73% (47/64) of organizations analyzed mentioned that consumers could use test results in their own diet and lifestyle decision making.

Over three-quarters (78%) of websites analyzed by Singleton et al [127] and about half of the websites analyzed by Lachance et al [121] and Lewis et al [122] mentioned limitations of test. None of the websites selling DTC-GT for thrombosis reported limitations [115].

Very little information or scientific evidence was provided on the clinical validity of tests [114-117,121-123,128]. Some websites referred to a laboratory certification, such as Clinical Laboratory Improvement Amendments (CLIA) standards, to indicate legitimacy [114,115,117,120-122,124,128].

### Scientific Evidence and Clinical Utility of Direct-to-Consumer Genetic Testing

A total of 14 papers, including two reports from the US Government Accountability Office (GAO) [11,129], question the scientific quality, clinical validity, and utility of DTC-GT (see Table 1). This issue was addressed in different ways.

**Table 1:** List of articles on scientific evidence and clinical utility of direct-to-consumer genetic tests.

Author	Aim of the study	Main findings
Adams, 2013 [130]	To investigate the reliability and reproducibility of DTC-GT by sending DNA samples to 2 popular companies	DNA samples from 2 individuals were sent to both companies. For 5 of 14 health conditions for which both companies reported relative risk information, the results were conflicting. The significance of relative risk changes was overemphasized, given that they were associated with very small changes in absolute risk.
Bloss, 2012 [131]	To evaluate the relationship between DTC genomic risk estimates and self-reported disease of individuals who went on to purchase a DTC-GT	For 5 out of 15 total conditions studied, the risk estimates from the test were significantly associated with self-reported family and/or personal health history.
Buitendijk, 2014 [132]	To explore the practicability and predictive value of DTC tests from four companies for age-related macular degeneration in 3 individuals	Predicted risks varied widely within each individual, and differences between highest and lowest estimates for lifetime risk were up to 12-fold. Within the same person, overall relative risks could be increased as well as decreased, depending on which test was used. None may represent the true disease risk.
Imai, 2011 [133]	To evaluate 3 DTC services and genomics service and compare the test results obtained for the same individual	The concordance rates between the services for single nucleotide polymorphism (SNP) data were >99.6%. There were some marked differences in the relative disease risks assigned by the DTC services due to different SNPs used to calculate risk for the same disease.
Janssens, 2008 [7]	To assess the scientific evidence supporting the purported gene-associations for genes included in genomic profiles offered online	The seven companies investigated tested at least 69 polymorphisms in 56 genes. Of the 56 genes tested, 24 were not reviewed in meta-analyses. For the remaining 32 genes, they found 260 meta-analyses that examined 160 unique polymorphism-disease associations, of which only 60 were found to be statistically significant. However the associations were modest.
Johnson, 2010 [134]	To survey potential notifiable variants on arrays used in genome-wide association studies and DTC genetic services	They identified 298 specific targeted mutations, encompassing 56 disorders. Only 88 out of 298 mutations could be identified as known SNPs in genomic databases. Eighteen out of 88 SNPs were found in commercially available arrays.
Kalf, 2013 [135]	To examine and compare the methods of 3 companies offering DTC-GT	Predicted risks differed substantially among the companies as a result of differences in the sets of SNPs selected and the average population risks selected by the companies, and in the formulas used for the calculation of risks.
Kido, 2013 [136]	To evaluate the distributions of disease risk prediction from three DTC companies using three Japanese samples	The overall prediction results were correlated with each other, but not perfectly matched; less than one third mismatching of the opposite direction occurred in 8 diseases of 22.
Mihaescu, 2009 [137]	To investigate the extent to which updating of risk predictions from commercial genome-wide scans leads to reclassification of individuals from below to above average disease risk or vice versa taking type 2 diabetes as an example	At individual level, 34% of 5297 participants switched between risk categories when risks were updated from 1-18 polymorphisms and 29% switched when age, sex, and body mass index were considered. In total, 39% of participants switched risk categories once and 11% switched twice.
Ng, 2009 [138]	To compare results of tests purchased from two DTC companies on 13 diseases for 5 individuals	For seven diseases, 50% or less of the predictions of the two companies agreed across 5 individuals.
Palomaki, 2013 [139]	To review the evidence about the clinical and analytic validity of type 2 diabetes genomic risk profiles promulgated by DTC-GT companies	The quality of evidence for analytic validity was inadequate. Clinical validity ranged from inadequate to convincing for 30 variants identified on five T2D genomic panels. Clinical utility evidence was inadequate.
Swan, 2010 [140]	To understand the variance in risk interpretation for multigenic conditions among 5 genome-wide DTC genomic companies	Multigenic condition risk interpretation may vary between DTC genomic services due to differences in the average lifetime risk assigned to similar underlying populations, the loci and SNPs selected for analysis, and the quantitative risk assignment methodologies used by DTC genomic companies.
Kutz, 2006 [129]	To evaluate the results of nutrigenetic tests purchased from four DTC companies for 14 fictitious consumers coming from two DNA samples	All 14 results predicted risk of developing different medical conditions. These predictions were similar for all the fictitious consumers, no matter which DNA or lifestyle description they used. One of the four companies gave contradictory results.
Kutz, 2010 [11]	To compare results from 10 tests each purchased from four DTC companies on 15 diseases for 5 individuals. To assess whether the tests provided any medically useful information	Each donor received risk predictions for the 15 diseases that varied from company to company. Four of the five donors received test results that conflicted with their factual medical conditions and family histories.

Seven studies [11,129,130,132,133,136,138] focused on the comparison of GT results from DTC companies for one or more individuals. The first study, executed by the GAO in 2006 [129], evaluated the results of nutrigenetic tests purchased from four DTC companies for 14 fictitious consumers with different characteristics obtained from two DNA samples. Interestingly, all 14 results predicted the risk of developing different medical conditions. These predictions were similar for all of the fictitious consumers, no matter which DNA or lifestyle description was used. Only one of the four companies gave contradictory results.

In a more recent report by GAO [11], 5 individuals purchased 10 tests manufactured by four different DTC companies. The tests were specific to 15 diseases. The analysis found a large variation in prediction risk from company to company. In agreement with the GAO report, Ng et al [138] found a modest concordance among the results (50% or less) from two DTC companies on 13 diseases for 5 individuals.

Similarly, Imai et al [133] compared the relative common disease risks obtained from three DTC-GT companies for the same individual and found comparable results from the single nucleotide polymorphism (SNP) analyses from different companies. However, in a similar recent study [132], they also pointed out a large variation in relative risks for some of the diseases investigated, possibly due to different SNPs used to calculate the same disease, the choice of the reference population, and the risk calculation methodology.

Bloss et al [131] compared the DTC genomic risk estimates with self-reported disease from individuals who purchased a GT. The risk estimates were significantly associated with self-reported family or personal health history in only five out of 15 conditions studied. Two studies [135,140] examined the risk assessment of common diseases in DTC-genomic services and found that the predicted risks differed among the companies due to different methodologies used, different loci, and SNPs selected for analysis.

In an evaluation of type 2 diabetes risk prediction from commercial companies offering genome-wide scanning [137], it was shown that the individual risk prediction changed depending on the number of polymorphisms used to calculate the risk and characteristics of people (eg, age and gender). In particular, 39% of 5297 individuals switched between risk categories once and 11% switched twice. A study by Palomaki et al [139] of type 2 diabetes, genomic risk profiles advertised by DTC-GT companies highlighted a lack of analytical validity and clinical utility in the tests through the Evaluation of Genomic Applications in Practice and Prevention Working Group. This approach was established to support the development of a systematic process for assessing the available evidence for GT in clinical practice.

Other studies focused on the scientific evidence of genetic polymorphisms used to estimate the disease risk by DTC companies. In particular, Janssens et al [7] looked for meta-analyses supporting 69 polymorphisms tested by seven companies and found inconsequential scientific evidence. Similarly, it was found that only 18 out of 88 SNPs identified as known SNPs in genomic databases associated with a disease were present in a commercially available test [139].

### Risk of Bias

The quality scores of the evidence reviewed ranged from 0.55-0.95. The majority of the studies that could be evaluated (69/80, 86%) had a score >0.7 (data not shown). Overall, all the studies are adequate in terms of methodological quality. The bias that was mostly present was a selection bias due to the recruitment of convenience population or a small sample size that did not allow a generalization of the results.

## Discussion

### Principal Findings

This review summarized the scientific literature on DTC-GT with a comprehensive view meant to unravel the complexity of the DTC-GT market. Previous systematic reviews dealt with this topic by focusing on certain aspects, particularly position statements, policies and recommendations [15], user perspectives [18], and health professionals’ perspectives [19]. This systematic review aimed to give an overall view of the DTC-GT market to include studies that analyzed the content of the websites offering these products, as well as studies focused on the scientific evidence and clinical utility of such tests. The large number of reports retrieved on this issue indicates a strong interest in the topic.

Thanks to the prevalence of the Internet over the past decade, the availability of health-related products on a DTC basis has become increasingly common. However, the fact that the promotion of these products such as drugs [141] or nutritional supplements [142] is comparable to the sale of any commodity is a cause for concern.

In terms of marketing, we must discuss the results considering the product, the offer, and the potential customers, in addition to the opinion of health professionals as product experts.

### The Product

Advances in genomic technology made GT available for both monogenic disorders and common complex diseases, in addition to nutrigenetic and pharmacogenetic tests. To date, the majority of these tests have provided a poor predictive value, and the assessment of the clinical validity and utility is still a work in progress. However, many commercial companies have begun to bring these tests to market. Their lack of scientific evidence was confirmed by some studies focused particularly on commercially available GTs [7,134]. Overall, all studies comparing the results of GT of the same people from different companies showed a modest concordance in risk predictions and sometimes reported contrasting results [11,129,130,132,133,136,138].

### The Offer

In general, the studies focused on the content of websites selling DTC-GT agree that these companies do not provide complete information to the consumer. This emphasizes the poor quality of information on the scientific evidence and clinical validity to support the tests. It also highlights the lack of attention to the risks related to the performance of the tests, genetic discrimination, emotional consequences, behavior changes, and confidentiality of test results. Furthermore, genetic counseling requirements were often missing. As with other sales-oriented companies, these websites contain marketing strategies that accentuate the benefits of the product. The benefits of testing were described more than the risks, and the theme of patient empowerment is highly emphasized as a good reason for testing [113,116,124,128]. The main emphasis on genes, without consideration for environment, might lead consumers to misinterpret test results, as was found by the majority of studies that addressed this issue.

### The Customer

Thirty-seven papers examined consumer knowledge of and attitude toward DTC-GT; likewise 37 papers discussed the impact of these tests on users. In general, it was confirmed that consumers have an interest in DTC-GT and that their main motivation is curiosity, as well as some interest in monitoring and improving health.

It should be noted that interest is highest among employees of biotechnology companies [30] or people at risk for cancer [35] and other diseases [25]—not the general public. Additionally, study participants were highly educated.

As for the impact of DTC-GT on users, nine papers and eight case studies were reviewed, in addition to those researched by Goldsmith et al [18]. The research remains limited because it evaluates the actual consequences of having a DTC test. Other studies reported situations where participants were exposed to mock reports about their genetic susceptibility or were asked to voluntarily submit to testing for research purposes. It seems that the feared negative consequences, such as psychological impact or increased anxiety for consumers, were not confirmed. Similarly, positive consequences, such as adoption of healthier lifestyle behaviors, were not observed, although a large proportion of people expressed the intention to change lifestyles [43,55,63,80].

Negative consequences may arise from misinterpretation of test results, which is another aspect addressed by several studies. The majority of studies showed that participants did not have particular difficulty understanding the test results. In fact, only a small number of people shared their results with a physician and reported worry after receiving them. Yet, it was also determined that the presence of a professional provided better interpretations of results compared to participants who received results online [87]. Interestingly, incorrect interpretation of results was confirmed in actual DTC-GT customers [43,91].

### The Expert

We considered health professionals’ perspectives as expert opinions, considering the strong recommendation for involvement of a health professional in the order process and interpretation of test results [143]. Compared to previous systematic reviews [19], 12 additional articles were retrieved that focused on health professionals’ perspectives and the overall scenario described by these authors were confirmed by our findings. As stated by Goldsmith et al [19], the level of awareness of DTC-GT remains inconsistent, even with three studies [41,100,110] in which the majority of physicians are aware of DTC-GT (but the sample size was ˂50 participants). In addition, few respondents have had direct experience with DTC-GT. The overall opinions regarding the utility of the tests are contrasting. Some professionals are in favor of some GT [99,101,102], while others considered it unacceptable to provide a predictive genetic test without genetic counseling [110] and were concerned about possible psychological harm [107], misunderstanding of results, and insurance discrimination [98]. Understandably, clinical geneticists expressed more concerns than general practitioners [107,110,111]. It should be noted that few physicians considered themselves confident in interpreting GT results and reported the need for more training [97,106,107,109]. In fact, an increase in the incorporation of instruction about application and technique in predictive genomic testing was presented in a recent study [103].

As discussed by some authors [97,144], these concerns raise the question of whether a non-geneticist physician involved in the commercial distribution of GT is properly equipped to offer test information to patients. So the presence of a physician does not guarantee the provision of adequate information. This is a cause for concern considering the recent shift from selling tests directly to the consumer to a direct-to-provider marketing model [97,118,144].

### Implications for Policy Making

We believe this review highlights the important aspects in considering the regulation of DTC-GT from a policy perspective. More specifically, there are at least three main issues to address to improve DTC-GT for a better service for the public:

1. DTC-GT is currently advertised despite the minimal and controversial nature of the supporting evidence. Here, more research is needed to evaluate these products and to eventually decide whether or not it is appropriate to market them at all.
2. As for other DTC products, GT is advertised by means of traditional strategies of persuasion generally used for commercial products (eg, more emphasis on benefits than on side effects). The rhetorical selling of DTC products calls for an enrichment of the guidelines for advertising of health-related products. In particular, these guidelines need to take into consideration the important literature from the fields of rhetoric and persuasion that explain how communication can be used to manipulate the beliefs and attitudes of consumers. The marketing of DTC-GT cannot be biased as it currently appears from the content analysis of websites.
3. From an ethical point of view, the first question to answer is whether, in light of the limitations in evidence and communication, DTC-GT empowers consumers. If empowerment is valuable because it is linked to autonomy, does current DTC-GT contribute beneficially to the development and application of autonomy? A second question concerns the fact that DTC-GT promotes products whose social implications have not been properly addressed. How does knowledge of self-assessed genetic risks influence the life of consumers? Current marketing of GT seems to be mainly interested in the advantages, as advantages convince consumers to purchase. But empowerment cannot be promoted separately from a full appraisal of the ethical aspects surrounding the delivery of a specific type of information.

Overall, research and practice must collaborate toward policy making in a field that is already open to the public despite its serious pitfalls.

### Limitations

Through a systematic approach we aimed to provide a comprehensive look at the DTC-GT market in order to better understand its actual impact on population. Although the number of articles retrieved is relatively large, some limitations related to studies design should be underlined. The majority of the studies used a cross-sectional design. It is known that response rate as well as sample size and sample selection are critical points in this kind of design [145]. Considering the total of the surveys investigating the awareness, use, and perceptions of health professionals and consumers, only 9 studies [30-33,40,52,56,59,81,92] out of 56 surveys (16%) (see Multimedia Appendix 2) have a response rate of at least 50% and a large sample size (more than 1000 subjects).

Most of the subjects recruited were highly educated and sometimes selection bias was present (eg, employees of health and technology companies [81]). All these aspects mean a poor representativeness of population [145]. Furthermore, few health professionals and consumers had direct experience with DTC-GT, so as previously argued [18,19], the responses of participants based on hypothetical scenarios make it difficult to draw conclusions about the actual impact of DTC-GT market.

### Conclusions

Based on the evidence collected, it seems that DTC-GT is neither beneficial nor detrimental to potential users. It should also be noted that the development of online companies is rapidly changing, most likely due to pressure from government agencies such as the FDA. Some companies have also changed their delivery model to include the health profession in the order process [143].

However, regardless of the large amount of data available on this issue, the actual experiences of DTC-GT users are still limited and this market is still in the early stages of distribution to the general public. Furthermore, some limitations on previous studies must be addressed. For instance, the majority of studies are characterized by people who do not represent the general public (participants were often convenience samples), featuring low sample size or limited response rate. Additionally, the prospective studies typically employ relatively short-term follow-up in the majority of the cases, not sufficient to evaluate the impact of DTC-GT on behavioral changes.

On the other hand, it is unacceptable that online companies offer GT lacking scientific evidence, no proven clinical utility, and misleading marketing claims. As underscored by Janssens and van Duijn [146], the expected benefits of whole genome scanning may be larger when tests are targeted only to specific at-risk populations, and not to populations-at-large, because of the moderate predictive ability of these current tests.

According to global industry analysts, the global genetic testing market is expected to reach more than US $230 million by 2018 [147]. Combined with the rapid decrease in biotechnology costs, this revenue stream will eventually allocate testing accessibility to all socioeconomic classes. It is important, therefore, to remain cautious and vigilant about this growing, influential health care market.

## Appendix

Multimedia Appendix 1 PRISMA checklist.

Multimedia Appendix 2 Supplementary tables.

## Abbreviations

BRCA: breast cancer susceptibility protein
DTC-GT: direct-to-consumer genetic testing
FDA: Food and Drug Administration
GAO: Government Accountability Office
GT: genetic testing/test
SNP: single nucleotide polymorphism
