# Supplementary material for: Internet-Based Direct-to-Consumer Genetic Testing: A Systematic Review
Source: J Med Internet Res. 2015 Dec 14;17(12):e279. doi: 10.2196/jmir.4378 (PMC4704942; doi:10.2196/jmir.4378)
Supplement: Multimedia Appendix 2 [file jmir_v17i12e279_app2.pdf]

Multimedia Appendix 2. Supplementary tables.

Table 1. List of articles on public's knowledge of and attitude to direct-to-consumer genetic tests.

Reference	Aim of the study	Number of participants	Response rate (%)	Characteristics of participants	Data collection method	Main findings
Adams, 2014 (US) [29]	To understand parents' experiences and knowledge related to a set neurodegenerative diseases of childhood onset for which no disease modifying therapies yet exist.	179	17	Recruited by the Batten Disease Support and Research Association; mean age (SD): 49.3 (11.7); 81% highly educated.[a]	questionnaire (quantitative)	73% felt that predictive genetic testing for an asymptomatic person should be available, regardless for medical benefit. Concerning DTC-GT, 82% of participants agreed or strongly agreed that people should always discuss GT plans and results with a professional, however the 32% also agreed that it should be possible for people to have GT on their own, without input from a doctor or genetic counselor.
Bloss, 2010 (US) [30]	To evaluate consumer perceptions of DTC personalized genomic risk assessments and assess the extent to which consumer characteristics may be associated with attitudes toward testing.	3640	74.5	Employees of large health & technology companies; mean age (SD): 46.2 (11.2); highly educated.[a]	online questionnaire (quantitative)	50% indicated that they had overall concern. In particular concerns regarded learning of disease risk (13%); unknown reaction to results (16%); quality and reliability of data (16%); privacy issues (36%). In general 82% would want to know their risk, no-one said definitely not.
Cherkas, 2010 (UK) [59]	To explore the reasons why people would consider taking personal genome testing and how they would use the genetic risk information provided.	4050	62	Recruited from Twins UK Adult Twin Registry; mean age: 56.	questionnaire (quantitative)	13% of respondents had heard of DTC-GT. 48% expressed interest in GT if the test was free of charge - Reasons for testing: 86% - to learn more about themselves; 80% -to convey risk to children, 79% to monitor health.

Critchley, 2014 (AUS) [51]	To assess the extent to which the public embrace DTC-GT relative to those obtained by a conventional medical practitioner (MP).	1000	16-21	Australian adults recruited as part of a national survey, mean age(SD): 56(16); 39% highly educated.[a]	telephone interview (quantitative)	People were significantly less likely to approve of, and order a DTC-GT offered by a company compared to that obtained by a MP.
Finney Rutten, 2012 (US) [31]	To assess population-level changes in awareness of DTC-GT in the US from 2008 to 2011.	7624 (2008) and 3959 (2011)	31 (2008) and 36.7 (2011)	Sampling from Health Information National Trends Survey representative of US adult population; aged 18-75+years; from 71% in 2008 to 84% in 2011 highly educated.[a]	mail and online questionnaire (quantitative)	Awareness increased significantly from 29% in 2008 to 37% in 2011.
Goddard , 2007 (US) [32]	To assessed consumers' awareness and use of nutrigenomic tests	5250	80	Sampling from HealthStyles survey; aged 18±65+years; 64% highly educated.[a]	questionnaire (quantitative)	14% of respondents were aware of DTC nutrigenomic tests and 0.6% used them.
Goddard, 2009 (US) [33]	To assess the public's knowledge of and interest in DTC nutrigenomic tests	Three samples:1867,2441,5499	from 52 to 63	Sampling from Behavioral Risk Factor Surveillance System survey; aged 18-65+years; from 18.5% to 58% highly educated.[a]	telephone survey (quantitative)	From 7.6% to 24% of participants were aware about DTC-GT. From 0.3% to 0.9% used a DTC-GT. Age, household income, and education level were consistently associated with awareness of DTC-GT for all of the surveys.
Gollust, 2012 (US) [34]	To assess the motivations, perceptions and intentions about personal genomics.	369	55.5	Recruited from the people who registered for the Coriell Personalized Medicine Collaborative; aged 18-65+years; 94%	online questionnaire (quantitative)	78% of participants were aware of personal genomics, 15% visited website for a DTC testing company, 2% purchased a DTC test. Motivations: curiosity (81%), finding out about their own disease risk and improving health (78%). Thirty per cent reported concern about test results. The 92% stated that they were likely to share their results with their physicians.

				highly educated.[a]		
Graves, 2011 (US) [35]	To explored interest in genetic testing for genes related to modest changes in breast cancer risk in women at moderate to high risk for breast cancer	105	84	Women aged 20-82 years; 64% highly educated.[a]; all with a family history of breast cancer, 23% affected with the disease.	telephone survey (quantitative)	77% of women indicated ‘definite’ interest in genetic testing. 63% did not understand relative vs. absolute risk. Women with less understanding reported more cancer worry and greater willingness to pay for testing.
Gray, 2012 (US) [36]	To evaluate the impact of risk information provision on women’s attitudes about DTC BRCA testing	767	90	Recruited online through Craigslist and The University of Pennsylvania's OncoLink Cancer Resource website; mean age (SD): 37(12).	telephone interview+online questionnaire (quantitative)	Women who were informed about the potential risks of DTC testing had lower intention to get BRCA testing (17% vs 24%, p=0.03), and less positive beliefs about DTC BRCA testing (72% vs 81%, P=0.01) than controls.
Hall, 2012 (US) [37]	To determine if awareness of, interest in, and use of DTC-GT is greater in sample of high-risk individuals, compared to controls.	1267	34	Recruited from the Northwest Cancer Genetics Network; mean age (SD) 62.4 (13.3); 55% highly educated.[a]	questionnaire (quantitative)	Overall 49% of respondents were aware of DTC-GT. Of those, 19% reported interest in using a DTC-GT and 0.9% used it. Awareness of DTC-GT was greater in sample of high-risk individuals than controls. However there were no statistical differences as regards the DTC interest.
Kaphingst, 2010 (US) [38]	To inform the ongoing debate over whether individuals offered DTC-GT can make informed decisions about testing when guided by online decision aids.	526	86	Recruited from members of a large health maintenance organization(#); mean age 34.6; 94% highly educated.[a]	telephone interview and website assessment (quantitative)	Regarding the Web-based genomic information presented using evidence-based communications, patients were more likely to describe their decision-making as easy (OR 1.04, 95% CI 1.01-1.07) and to decide to be tested (OR 1.08, 95% CI 1.05-1.11).

Kolor, 2012 (US) [39]	To explore awareness and use of DTC-GT in four USA states.	Four samples: 6019, 5883, 1931, 2606.	44,57,42,67 respectively	Sampling from Behavioral Risk Factor Surveillance System survey; aged 18±75+years; from 40% to 67% highly educated.[a]	telephone interview (quantitative)	From 16% to 29% of participants were aware about DTC-GT. From 0.5% to 0.8% used a DTC-GT. Age and high education level were consistently associated with awareness of DTC-GT for all of the surveys.
Langford, 2012 (US) [40]	To examine the association of race/ethnicity and numeracy with awareness of DTC-GT.	6754	100	Sampling from Health Information National Trends Survey; aged 18±75+years; 39% White,25% Black, 19% Hispanic highly educated.a	telephone interview (quantitative)	Black respondents were generally less aware on DTC-GT than white respondents (OR 0.79, 95% CI 0.65-0.97). People with highest level of education had significantly greater knowledge of DTC-GT than people with less than a high school education (OR 2.43, 95% CI 1.89-3.12).
Leighton, 2012 (US) [41]	To investigate consumers' perceptions and understanding of DTC test results.	145	39	Facebook users; mean age 28.1; 98% highly educated.[a]	online questionnaire using facebook (quantitative)	The 48% of the participants did not have familiarity with DTC-GT. The study demonstrated that the general public is at risk for misunderstanding DTC genetic test results if left to interpret them on their own. Overall, 92 out of 106(87%) respondents stated that they would seek more information from their doctor.
Lynch, 2011 (US) [26]	To identify common themes, attitudes, and ethical concerns in the US news media coverage of DTC-GT.	92 stories	NA	NA	Analysis of stories on DTC-GT (qualitative)	Stories displayed moderate genetic determinism and were neutral about validity and utility. They indicated that insurance and employers were the most likely sources of discrimination, yet identified the physicians and DTC companies as groups most likely to violate privacy. Stories claimed lack of regulation would harm consumers. The costs of test were frequently included.
Mai, 2011 (Greece) [52]	To evaluate the awareness of the general public and physicians on the genetic testing services in Greece.	1717	100	Recruited from general public selected from four major cities in Greece; 50% aged 35-60 years.	questionnaire (quantitative)	Awareness of public and private GT laboratories was 68%. Among the general public the 18% was in favor of DTC-GT.

McBride, 2009 (US) [42]	To evaluate what psychological and behavioral factors predict who is likely to seek online personalized genomic risk.	1959	33	Recruited from members of a large health maintenance organization; mean age 35; 75% highly educated.[a]	online questionnaire (quantitative)	Of those who viewed the information pages on the Web site, 50% considered the pros and the cons of free testing, and decided not to be tested. Those considering genetic susceptibility testing did hold genetically deterministic beliefs, but believed genetic information to be valuable.
McGuire, 2009 (US) [43]	To explore social networkers' interest in and attitudes toward DTC-GT, focusing on expectations related to the clinical integration of GT results	1087	23	Facebook users; mean age(SD): 35(12); 91% highly educated.[a]	online questionnaire using facebook (quantitative)	Of all respondents 47% heard of DTC companies. 64% would consider DTC-GT in future; 30% would not use them, 6% purchased them. 34% consider the information obtained from DTC-GT to be a medical diagnosis; 81% reported curiosity about genetic-make-up as the main reason for testing and 78% of those who would consider DTC-GT would ask their physician for help interpreting test results.
Nielsen, 2012 (CAN) [57]	To conduct a randomized control trial to assess behavioral outcomes as well as the awareness, perceptions, and understanding of nutrigenomics and genetic testing.	149	95	Recruited from the Toronto Nutrigenomics and Health Study; mean age(SD) 26(4) years; 80% highly educated.[a]	online questionnaire (quantitative)	Of respondents 52% reported having heard ‘‘nothing’’ about DTC genetic testing, 46% were interested in the relationship between diet and genetics, 66% strongly agreed that they would benefit from learning about how their genetic makeup affects their diet.<br>Only 9% agreed that they felt uneasy learning about their genetics. - 88% (intervention group) vs72% (controls) considered useful the recommendations on their diet (p=.009)
O'Neill, 2008 (US) [44]	To examine the feasibility of offering genetic susceptibility testing for lung cancer via the Internet to smokers who had a family history of lung cancer.	304	56	116 eligible relatives expressed further interest in receiving information online. Mean age (range): 38(20-54); 68% highly educated.[a]	telephone interview (quantitative)	Participants who logged on expressed greater quit motivation (OR=1.7), awareness of GT for cancer risk (OR=3.1) and were more likely to have a daily Internet access (1.4) than those who did not log on. Forty-eight percent of those who logged on made informed decision to be tested.

Ortiz, 2011 (Puerto Rico) [45]	To determine the prevalence and correlates of awareness of DTC-GT and the prevalence of genetic test use in a population-based sample of adults in Puerto Rico.	611	96	Sampling from Health Information National Trends Survey in Puerto Rico; mean age (SD) 25 (4); 45% highly educated.[a]	telephone interview (quantitative)	56% of respondents were aware of DTC genetic tests
Paquin, 2012 (US) [46]	To examine the association of individual dispositional differences with health risk perceptions and online information seeking related to a free GT.	1959	30	Recruited from members of a large health maintenance organization; mean age 35; 75% highly educated.[a]	telephone interview (quantitative)	Neuroticism, a dispositional characteristic commonly linked to adverse emotional response, was not predictive of online information seeking (OR 1.03, 95% CI 0.97-1.10).
Perez, 2011 (US) [47]	To examine women's attitudes towards DTC advertisements and online testing for BRCA.	84	97.7	Convenience sample of women who had received genetic counselling and at increased risk for breast and ovarian cancer on the basis of family history; mean age (SD): 55 (10); 91% highly educated.[a]	online questionnaire (quantitative)	The 73% of women heard about genetic testing; 59% had positive attitude toward DTC advertising however 74% had negative attitudes toward the availability of online genetic tests; 46% strongly agreed that it is more appropriate for companies to target doctors to identify women who may be at risk for carrying the breast cancer gene than target all women through media.
Rahm, 2012 (US) [27]	To determine knowledge, attitudes and beliefs about DTC-GT among members of a large managed care organization, and whether differences might exist between	39	>50%	Randomly selected from Kaiser Permanente Colorado members populations adults; aged 30-60+ years; 79% highly educated.[a]	questionnaire to focus groups (qualitative)	About the 45% of participants were negative towards DTC genetic testing and advertising without differences among focus groups.

	population groups.					
Ramakrishnan, 2011 (US) [48]	To understand consumer's responses attitudes, behavioral intentions and information seeking behavior in response to a fictitious DTC-GT advertisement	410	NA	Aged 18-65+ years; 53% highly educated.[a] Selected from an online survey.	online questionnaire (quantitative)	Only 11% of respondents had ever seen advertisement for a predictive genetic test. Consumers reported moderate intentions to talk with their doctor and seek more information about DTC-GT after advertisement exposure (50%). The 32% of the respondents exhibited intentions to take the test.
Ries, 2010 (CAN) [53]	To explore attitudes and interest regarding different types of genetic tests	1211	NA	Selected among adult residents of a Province of Alberta, Canada through a telephone survey; aged 18-65+ years; 75% highly educated.[a]	telephone interview (quantitative)	Nearly 70% of respondents were unaware about DTC-GT. Sixty-one percent of respondents stated that the availability of treatment for a genetic condition would have a strong influence on their decision to undergo testing. However the 37% stated that they would pay nothing for GTs related to a manageable condition.
Rollins, 2014 (UK) [61]	To evaluate consumer perceptions, intentions and behavior associated with DTC-GT advertising.	410	60	Recruited through Qualtrics online survey software; aged 18-65 years; 42% highly educated.[a]	online survey (quantitative)	Only 11% of the respondents had ever seen an advertisement for a DTC-GT.<br>Overall, consumers reported moderate intentions to talk with their doctor and seek more information about GTs after advertisement exposure.<br>Those who perceived greater threat from the disease had significantly greater behavioral intentions to talk to their doctor and search for more information about the test. However, it did not affect consumers' intentions to take the test.
Sanderson, 2008 (UK) [54]	To explore anticipated reactions to genetic test results and interest in genetic testing for two chronic	1024	51	Selected randomly from a primary care register of 8000 adults in Oxfordshire, England; aged 18-	questionnaire (quantitative)	Interest for genetic testing was 78% for cancer risk and 80% for heart disease risk. The major interest was expressed by respondents who anticipated to be glad to know their genetic risk, in particular 72% of who received cancer risk test and 82% of who received heart risk disease test.

	conditions - cancer and heart disease.			75 years; 46% highly educated.[a]		
Savard, 2014 (AUS) [58]	To explore the knowledge and perceptions of DTC-GT in an Australian sample.	270	70	Aged 18-31 (56%); 79% highly educated. Selected from an online survey	online questionnaire (quantitative)	Only 40% of respondents correctly identified that DTC-GT were available in Australia.<br>Only 27% of the respondents expressed an intention to pursue the GT. However the 43% indicated the information provided by DTC-GT medically, personally and at family level important.
Sweeny, 2011 (US) [49]	To examine predictors of interest in DTC-GT	99	39	Recruited through web advertisements for the study; mean age 37.3; 93% highly educated.[a]	online questionnaire (quantitative)	Intentions to pursue DTC-GT were significantly correlated with perceived benefits, perceived barriers, anticipated regret over testing and not testing, $r>0.39$, $p<0.001$.
Tercyak, 2011 (US) [50]	To describe parents' attitudes toward pediatric genetic testing for common, adult-onset health conditions and to identify underlying these attitude.	219	92.4	Recruited from members of a large health maintenance organization; mean age (SD) 35.2 (3.6); 79% highly educated.[a]	online questionnaire (quantitative)	Variables associated with decision balance regarding GT included greater interest in knowing about gene-health associations in their child and more positive emotional reactions to learning about their child's decreased risks ($R^2=0.33$; $p<0.001$)
Vayena, 2014 (Switzerland) [55]	To examine the attitude of Swiss University students to DTC-GT	1146	5.6	The participants were recruited among a large sample of University students; mean age 25; All highly educated.[a]	online questionnaire (quantitative)	The 59% of the respondents were interested in DTC-GT<br>The key reasons for potentially undergoing the testing were to contribute to scientific research (72%) and to receive health information (66%)
Vermeulen, 2013 (The Netherlands) [60]	To determine public attitudes and interests towards offering GT and family-based risk assessment for	978	70	A representative group of a national survey (Dutch Health Care Consumer Panel); mean age 59 (range	paper or online questionnaire (quantitative)	About half of the respondents expressed an interest in GT to prevent common diseases; in particular those lower-educated.<br>Half of the respondents believed that family history assessment could help prevent diseases, but only 21% thought family history should be

	common chronic disease prevention.			18-91); 33% highly educated.[a]		assessed for everyone.
Wasson, 2012 (US) [28]	To assess the attitudes and views, decision-making factors and ethical considerations of primary care patients about DTC-GT.	29	NA	Primary care patients recruiting from the waiting room at an urban, academic medical center clinic; mean age 51; 65% highly educated.[a]	Focus group interviews (qualitative)	Participants reported that they were motivated to test by a desire for information about themselves and potential prevention of disease. Some concerns regarded the accuracy of test and interpretation of results.
Wilde, 2010 (AUS) [25]	To qualitatively assess public understanding of, and attitudes towards, risk prediction involving susceptibility genes for depression.	36	NA	Recruited from a market research database; mean age 41; 47% high school.	Focus group interviews (qualitative)	Of participants, 71% of affected by a depression disease and 64% of unaffected indicated interest in having a genetic test for susceptibility to major depression. Participants were unanimously against online DTC-GT.
Wilde, 2011 (AUS) [56]	To test Interest in predictive testing for a depression risk genotype.	1046	68	Nationally representative sample of Australia from computer-generated list of phone numbers; mean age 51; 55% highly educated.[a]	telephone interview (quantitative)	The study found strong interest in predictive genetic testing for a reported susceptibility to depression. However there was significantly greater interest in seeking such a test through a doctor (63%) compared to DTC (40%) ($p<0.001$).

AUS: Australia; DTC-GT: direct to consumer-genetic test; CAN: Canada; NA: Not available; UK: United Kingdom; US: United States.

[a]College degree or more.

Table 2. List of articles on the impact of direct-to-consumer genetic tests on users.

Reference	Aim of the study	Number of participants	Response rate (%)	Characteristics of participants	Actual DTC-GT consumers	Study design	Duration of follow-up (months)	Main findings
Bansback, 2012 (CAN) [80]	To determine the feasibility of eliciting individuals' interpretations of different hypothetical genetic risk profiles.	319	69	Recruited by email from a market research panel; median age 53 years; 65% highly educated.[a]	no	descriptive (quantitative)	-	SHARING RESULTS: Most participants anticipated addressing a doctor (63%), (BEHAVIOR) lifestyle changes (57%) and, accessing screening (57%). WORRY: 40% anticipated feeling more worried an anxious. RESULTS UNDERSTANDING: 1% found results difficult to understand.
Bloss, 2011 (US) [81]	To examine the psychological, behavioral, and clinical effects of risk scanning with a commercially available test.	2037	56% completed 3 months of follow-up	Employees of large health & technology companies; mean age(SD); 46.7(12.0) years; highly educated.[a]	no	prospective (quantitative)	3	WORRY: No significant differences between baseline and follow-up in anxiety symptoms and lifestyle behaviors and 90% of subjects indicated no significant test-related distress. SHARING RESULTS: A total of 10% of subjects reported discussing their results with the company genetic counselor, and 27% with their physician.
Bloss, 2013 (US) [82]	To determine the long term psychological, behavioral, and clinical impacts of genomic risk testing for common disease.	1325	36% completed 1 year of follow-up	Recruited as previous paper (Bloss et al, 2011); mean age (SD); 47.5 (12.2) years.	no	prospective (quantitative)	12	WORRY AND BEHAVIOR: No significant differences between baseline and follow-up in anxiety symptoms and lifestyle behaviors and 97% of subjects indicated no significant test-related distress. SHARING RESULTS: A total of 40% of subjects reported discussing their results with their physician. RESULTS UNDERSTANDING: The majority of individuals reported the comprehension of their results (75%) and considered them very useful (62%).

Bloss, 2014 (US) [83]	To evaluate the actual impact of DTC pharmacogenomics testing on consumer behavior.	1325	36% completed 1 year of follow-up	Recruited as previous paper (Bloss et al, 2011).	no	prospective (quantitative)	12	WORRY AND BEHAVIOR: DTC pharmacogenomics testing risk profiling among a selected sample of individuals was associated with increased physician utilization and did not result in any adverse changes in psychological health or follow-up test-related distress.
Boeldt, 2014 (US) [93]	To investigate the influence of individual differences in disease perceptions among consumers on testing outcomes.	2037	56%	Recruited as previous paper (Bloss et al, 2011).	no	prospective (quantitative)	3	WORRY AND BEHAVIOR: The combination of high-perceived seriousness and high-risk genetic result for a feared disease was associated with an increase in genomic test-related distress, but not clinically significant. In contrast, no significant associations were observed for diet, exercise and screening behaviors.
Dar-Nimrod, 2013 (US) [84]	To assess affective, attitudinal, motivational, and behavioral effects of simultaneously learning about one's own and other's genetic susceptibility to alcoholism.	160	NA	Participants arrived at meeting place at hospital to take part in a study on genetics; mean age (SD): 20.5 (3.8) years.	no	descriptive (quantitative)	-	WORRY: It was shown a significant increase in negative effect among individuals who learned that they have susceptibility to alcoholism. BEHAVIOR: Intentions for alcohol consumption in the near future were not affected.
Darst, 2013 (US) [85]	To assess DTC-GT consumers' utilization and perceptions of genetic counselling services.	1325	36% completed long term follow-up	Recruited as previous paper (Bloss et al, 2011); aged 19-84 years; 25% highly educated.[a]	no	prospective (quantitative)	14 months	SHARING RESULTS: 14% had spoken with a genetic counselor. The most commonly reason for not utilizing the counselling service was the perception of understanding one's results (56%). The 14% already consulted their physician.
Darst , 2014 (US) [86]	To evaluate the characteristics of DTG-GT consumers who spontaneously shared their test results with their health care provider.	2024	56% completed short-term follow-up	Recruited as previous study (Bloss et al, 2010); 540 health care provider sharers versus 1484 non-sharers; mean age(SD): 50(12.5) years	no	prospective (quantitative)	6 months	WORRY: 45% sharers vs 53% non-sharers (p = .01) reported overall concerns related to testing. 11% sharers vs 13% non-sharers (p = .2) reported concerns about learning disease risk. RISK PERCEPTION: Greatly value attributed to risk information in 78% sharers and 69%

				sharers and 45(11.6) non-sharers				non-sharers (p < .01)
Egglestone, 2013 (UK) [94]	To examine consumers' perceptions of the effect of genetic risk information, provided by DTC-GT, on their health behavior and health anxiety.	189	69% purchased DTC-GT	Recruited through social media; aged 18-60+ years; 92% highly educated.[a]	yes	descriptive (quantitative)	-	BEHAVIOR: 27% of consumers claimed a change in health behavior, particularly healthier diet (53% of them). Consumers had significantly better health behavior scores than potential consumers (n=86) (p = .02). WORRY: 25% declared a change in health anxiety after receiving results. RISK PERCEPTION: No significant correlation was found between perceived risk and health behavior, general anxiety about health or about developing serious disease.
Francke, 2013 (US) [90]	To evaluate benefits and harms experienced by consumers who purchased a DTC-GT.	62	21	Recruited from 23andMe customers database; 32 mutation-positive "cases" and 31 mutation-negative "controls"; mean age: 47 years	yes	descriptive (quantitative)	-	BEHAVIUOR: 25% of cases (50% of women) had diagnostic or therapeutic interventions and 44% of those women planned breast therapeutic interventions. WORRY: Among the "cases" 28% felt anxious only at first and 13% felt moderate anxiety; 53% were neutral. SHARING RESULTS: 60% of the cases and 26% of controls reported to have shared test results with their physician.
Goddard , 2007 (US) [32]	To assessed consumers' awareness and use of nutrigenomic tests.	29	80% (referred to the all sample of 5250 respondents)	Sampling from HealthStyles survey; aged 18-65 years; 69% highly educated.[a]	yes	descriptive (quantitative)	-	Test users were more likely to have a positive family history for most of the diseases that were investigated including heart disease (59%) and diabetes (48%) compared to the whole sample. SHARING RESULTS: 10% discussed their test results with a health care provider.

Gordon, 2012 (US) [70]	To evaluate the impact of disease risk assessment on health behavior.	60	NA	Participants to Coriell Personalized Medicine Collaborative study; mean age: 48.9 years, 60% highly educated.[a]	no	prospective (qualitative)	3	WORRY: The majority of participants (88%) reported to reassured or accept test results but the 25% reported also being worried about them and 25% shared them with a health care provider. BEHAVIOR: About one-third reported making a change in their lifestyle. RESULTS UNDERSTANDING/RISK PERCEPTION: The 67% of participants appeared to understand the multi-factorial nature of common complex disease.
Haga, 2014 (US) [87]	To address the effectiveness of in-person versus online delivery models for understanding genomic risk and adoption of healthy behaviors in people tested for type 2 diabetes mellitus risk.	300	80% completed the 3 months follow-up	Non-diabetic participants recruited from Duke University and surrounding areas; aged 18-29 years (44%), 91% highly educated.[a]	no	prospective (quantitative)	3	UNDERSTANDING/RISK PERCEPTION: Participants who received their results in-person were more likely than those who reviewed their results online to correctly interpret their genomic risk (72 vs.47%, $p < 0.001$) and report their actual genomic risk (69 vs. 49%, $p < 0.001$). WORRY: At 1-week follow-up, the 92% of participants expressed no regret after receiving their test results.
Harris, 2014 (UK) [62]	To examine videos posted by users of DTC-GT services in order to understand more about how people are engaging with this technology.	20	-	Videos uploaded by users of America company 23andMe	yes	descriptive (qualitative)		From the analysis of stories telling by DTC-GT users emerged a sense of indifference toward the results of the GT. In general the engagements with disease and disease risk seem fleeting and playful.
James, 2011 (US) [88]	To assess the impact of DTC predictive genomic risk information on perceived risk and worry in the context of routine clinical care.	150	43	74 received DTC-GT plus usual care; 76 received usual care alone; aged 40-69 years; highly educated.[a]	no	RCT	12	RISK PERCEPTION: Participants who were tested perceived risk information initially rated higher for 4 conditions compare to the others ($p < .05$). WORRY: 34-54% worried about cancer disease; 81% worried about myocardial infarction (similar to controls). At 1 year there were no differences between groups.

Kaphingst, 2012 (US) [89]	To examine patients' responses to DTC-GT and their interpretation of test results.	199	NA	Recruited from members of a large health maintenance organization; mean age (SD): 35 (4.2) years, 52% highly educated.[a]	no	prospective (quantitative)	3	WORRY: On average, test feedback generally did not evoke strong positive (means 3 to 4) or negative psychological responses (means 1 to 2.6) on a scale of 1-7, 1 indicating "not at all") RISK PERCEPTION: Testers did not generally interpret test results as deterministic of health outcomes (mean=6.0 on a scale of 1-7, 1 indicating strongly deterministic) - Only 1% reported having talked about their results with an health-care provider.
Kaufman, 2012 (US) [91]	To measure customers' opinions 2 to 8 months after they had received their results.	1048	37	Customers of three DTC-GT companies who received their results 6 to 28 weeks prior the invitation; aged 18-55 years; highly educated.[a]	yes	descriptive (quantitative)	-	BEHAVIOR: 16% changed a medication or supplement regimen and 33% played more attention on diet. SHARING RESULTS: 28% had discussed their results with a health care professional. RISK PERCEPTION: 25-45% reported incorrect interpretations of two sample risk results.
McGowan , 2010 (CAN) [67]	To investigate early users' reasons for utilizing personal genome services.	23	NA	Recruited by using the Google search engine and entering key words to capture people using blogs associated with DTC companies websites; mean age 42 years.	yes	descriptive (qualitative)	-	BEHAVIOR: 83% of participants reported no influence of their test results on preventive health actions. SHARING RESULTS: 13% consulted their physician for preventive measures advice. RISK PERCEPTION: Many respondents reported that DTC tests have low predictive power and no problem in interpreting the results. REASONS: The two most commonly cited reasons for having a personal genome scan were 1) to gain health-related information and 2) to learn about individual genetic risk factors.

McGuire, 2009 (US) [43]	To explore social networkers' interest in and attitudes toward DTC-GT, focusing on expectations related to the clinical integration of GT results.	63	23% (referred to the all sample of 1087 respondents)	Facebook users; mean age(SD): 35(12); 87% highly educated.[a]	yes	descriptive (quantitative)	-	BEHAVIOR: 75% declared that the GT-results would influence their future health care decisions and modify lifestyle. SHARING RESULTS: 53% have discussed results with physician. UNDERSTANDING RESULTS/RISK PERCEPTION: 43% declared to understand the results, however 60% considered information obtained from DTC-GT to be diagnosis of medical condition. REASONS: The main reason reported by the 81% of users was the general curiosity about genetic make-up.
Nielsen, 2014 (CAN) [95]	To conduct a randomized control trial to determine whether a personalized nutrition intervention modifies perceptions.	125	80	Recruited from the Toronto Nutrigenomics and Health Study; 83 received dietary advice based on genotype; 42 received general dietary recommendations; mean age (SD) 26 (4) years; 80% highly educated.[a]	no	RCT	12	PERCEPTION: certain perceptions of personalized nutrition and genetic testing changed over the course of the study, although these changes do not appear to be result of providing DNA-based dietary advice.<br>SHARING RESULTS: A significantly greater proportion of participants in the intervention group, shared the information with a family member (31 vs 9%, p=.0.005) or a friend (64 vs 10%, p<.0.01). Only 1 participant (Intervention group) shared the results with a health care professional.
Nusbaum, 2013 (US) [63]	To explore attitudes about and initial responses to genomic testing for colon cancer risk.	20	63	Recruited from a Division of General Internal Medicine. Mean age (SD): 61 (11) years; 85% highly educated.[a]	no	descriptive (qualitative)	-	BEHAVIOR: Al least 50% of the sample discussed making dietary and exercise changes. SHARING RESULTS: At post-test 30% reported that they would discuss SNP results with their doctors. WORRY: None of participants expressed anxiety or distress during post-test session or interview. RISK PERCEPTION: Most participants perceived their SNP risk profile as indicating slightly increased risk for colon cancer relative to their risk perception prior to the study.

Reid, 2012 (US) [92]	To examine whether offers of multiplex genetic testing increases health care utilization among healthy patients aged 25-40.	1599	62	Recruited from members of a large health maintenance organization; mean age: 34.9 years, 76% highly educated.[a]	no	descriptive (quantitative)	12	BEHAVIOR: No changes in overall use of health care among those receiving personalized genetic test results compared to those who were not tested. (GT users=217)
Su, 2011 (Belgium) [68]	To explore the motivations and expectations of people who have used DTC-GT.	56 (stories obtained included at first analysis)	47	Stories of customers who purchased a DTC-GT, extracted from blogs on DTC and other websites.	yes	descriptive (qualitative)	-	REASONS: The major motivations and reasons for purchasing DTC-GT are related to health.
Su, 2013 (UK) [69]	To perform a content analysis of comments written by individuals who signed a public online petition initiated by DIYgenomics (CA, USA) to support “personal access to genetic information”.	247	NA	Comments written by individuals who signed a public online petition initiated by DIYgenomics (CA, USA) to support “personal access to genetic information”.	yes	Descriptive (qualitative)	-	Among the reasons raised by petitioners in support of unrestricted access to DTC GT there were: that their ownership of their DNA should allow them to have unrestricted access to their genomic information; that they should have the right to their genomic information; that the government has no place in (further) regulating DTC GT; that health care professionals should not be placed as intermediaries when purchasing DTC GT services; that many petitioners who had already obtained DTC GT had positive experiences with this model of provision.
Vayena, 2012 (Switzerland) [71]	To explore attitudes, motivations and self-reported impact in connection with DTC-GT amongst a group of life scientists.	40	67	Researchers and students attending graduate courses in molecular genetics, aged 25-40 years; all highly educated.[a]	no	descriptive (quantitative)	-	WORRY: 43% expressed no concerns at all. SHARING RESULTS: 28% intended to show results to a health practitioner RISK PERCEPTION: 35% declared the results had no impact on their health perception.
		10	100		no	prospective (qualitative)	5	BEHAVIUOR: All participants said that the results did not have a significant impact on their lives or their attitude towards their health. SHARING RESULTS: Most of respondents found

								unnecessary discussing results with a physician.
Vayena, 2014 (Switzerland) [55]	To examine the possible reactions to disease risk probabilities of University students exposed to two hypothetical results indicating high and low risk of colon cancer and obesity.	1146	5.6	The participants were recruited among a large sample of University students; mean age 25; All highly educated.[a]	no	online questionnaire (quantitative)	-	RISK PERCEPTION: Reactions to mock results showed that increased colon cancer risk would likely generate more action (53%) including involving a medical doctor (50%). Also in the case of an increased risk of obesity the most commonly reaction was that of taking preventive measures (48%). WORRY: The concern about the results was reported by the 39% and 11% of students in the case of increased colon cancer risk and obesity, respectively.
Vernez, 2013 (US) [64]	To investigate student experiences and attitudes towards DTC-GT.	10	NA	Students who chose to undergo personal genotyping in the context of a genetic course, over 18 years old. All highly educated.[a]	no	prospective (qualitative)	6	BEHAVIUOR: students did report intentions to make modest changes as a result of risk predictions of complex diseases or conditions. However they did not report making significant progress on these intentions 6 months after receiving test results. SHARING RESULTS: Students did not report utilizing genetic counselling.
Wasson, 2013 (US) [65]	To examine the views, attitudes and decision-making factors of primary care patients regarding DTC-GT	20	NA	Primary care patients recruiting from the waiting room at an urban, academic medical center clinic; mean age 50; 70% highly educated.[a]	no	prospective (qualitative)	12	WORRY: Most participants did not report specific concerns. BEHAVIOR: Most participants claimed that testing had no impact on them over time, however many of them viewed their disease risk estimates as not worrying or bad.

Wasson, 2014 (US) [66]	To investigate the factors associated with disclosure decisions regarding DTC-GT.	20	NA	Recruited as previous paper (Wasson et al, 2013).	no	prospective (qualitative)	12	SHARING RESULTS: Most participants disclosed to some immediate family; less than half disclosed to extended family; approximately half talked to friends. Most participants stated that they would or might disclose to physicians about DTC-GT and few did.<br>RISK PERCEPTION: Few participants reported finding their results clinically useful.

CAN: Canada; DTC-GT: direct to consumer - genetic test; RCT: randomized control trial; UK: United Kingdom; US: United States.

[a]college degree or more

Table 3. List of articles on health professionals' point of view on Direct-to-consumer genetic tests.

Reference	Aim of the study	Characteristics of participants	Response rate (%)	Study design	Main findings
Bernhardt, 2012 (US) [98]	To assess the responses of primary care physicians to reports of DTC-GT presented by a hypothetical patient, and their attitudes toward personalized genomics.	315 family practitioners and 187 internists	23	online survey (quantitative)	EXPERIENCE: 50% ordered GT more than once a year and only 16% ordered tests one a week or more. The 7% indicated that they had previously seen a patient's DTC genetic risk assessment report. The 16% were very confident in interpreting GT results. OPINION: More than 80% as regards pharmacogenomics report and more less than 80% as regards disease risk report agreed that the report was understandable. The 70% in the first case and the 40% in the second one believed the results would be helpful in managing the patient and therefore clinically useful. As regards genomic testing about 47% agreed that GT would motivate patients to adopt healthy BEHAVIOR. CONCERN: 68% reported being concerned about a possible insurance discrimination.
Brett, 2012 (AUS) [109]	To explore genetic health professional's opinions of DTC-GT and reported frequency of individuals presenting to clinical genetics services after undertaking testing.	130 genetic counsellors (GCs); 38 clinical geneticists (CGs)	44	online survey (quantitative)	EXPERIENCE: 11% reported having had clients consult them after undertaking DTC-GT. Only 7% were confident in interpreting and explaining DTC-GT results. OPINION: the majority of respondents (54%) didn't consider DTC-GT useful for individuals.
Brierley, 2014 (US) [108]	To explore what genetic counselors and physicians from non-genetics specialties would do if faced with complex decisions associated with cancer genetic testing, how their views compare, and how they align with current guidelines and data.	216 cancer GCs; 1097 non-genetics physicians	NA	online survey (quantitative)	OPINION: GCs were less likely compared to non-genetics physicians to test their minor children (0.9 vs 33.1%; $p < .001$) or test their children without their knowledge and consent/assent (1.4 vs 11.5%; $p < .001$).<br>GCs choices are more aligned with professional guidelines.
Giovanni, 2010 (US) [99]	To ask specialists in clinical genetics about their experience with individuals who consulted them after DTC-GT	121 GCs; 6 medical geneticists and 6 health-care providers	3	online survey (quantitative)	EXPERIENCE: 17% reported providing consultations to patients undertaking DTC-GT. OPINION: 52% of providers described the test as clinical useful particularly referring to BRCA1/2 testing (breast cancer susceptibility) (86%).

Goddard, 2007 (US) [32]	To assess knowledge of and experiences with DTC-nutrigenetic tests among US physicians.	1250 physicians practicing in the US	61	online survey (quantitative)	EXPERIENCE: 44% reported they were aware of DTC nutrigenetic tests and 11% reported their patients had asked about these test.
Haga, 2011 (US) [100]	To assess physician attitudes and uptake of genomic risk profiling among an early adopter practice group	157 primary care physicians	44	online survey (quantitative)	AWARENESS: 90% of respondents had heard of genomic risk profiling offered by online DTC companies. EXPERIENCE: 42% had ordered a GT for a patient and one third for themselves. OPINION: 67% of respondents who did not order GT for their patients expressed concern about clinical utility and 58% of those who did it.
Hock, 2011 (US) [101]	To assess GCs' experience, knowledge, and beliefs about DTC-GT	312 member of the National Society of GCs	36	online survey (quantitative)	EXPERIENCE: 46% of respondents had worked with patients about DTC-GT. Only 15% had suggested them to patients. OPINION: 51% of respondents thought GT should be limited to a clinical setting; 56% agreed DTC-GT is acceptable if genetic counseling is provided. Respondents were more likely to agree with statements regarding potential DTC risks that DTC benefits.
Howard and Borry, 2013 (EUROPE) [110]	To estimate the awareness of, experiences with, and attitudes towards DTC-GT of European clinical genetistics.	131 clinical genetistics from 28 European countries.	44	online survey (quantitative)	AWARENESS: 86% had heard about DTC-GT. EXPERIENCE: 44% had been contacted by at least one patient who asked about the DTC_GT after having purchasing a test. OPINION: The majority (86%) of respondents considered totally unacceptable to not provide face-to-face medical supervision for a predictive test for a condition that has a "medium" penetrance of 50 to 60%. All respondents expressed the unacceptability of offering DTC-GT for conditions neither treatable nor preventable.
Leighton, 2012 (US) [41]	To investigate consumers' perceptions and understanding of DTC-GT results relating to four common disease risks, compared to GCs.	171 member of the National Society of GCs	10	online survey (quantitative)	The general public respondents believed that results from the DTC-GT would be significantly more helpful in managing future medical care than the GCs. AWARENESS: 95% had heard about DTC-GT.
Mai, 2011 (Greece) [52]	To learn about how medical practitioners perceive GT.	496 physicians from all medical specialties who attended a Greek medical conference.	NA	survey (quantitative)	OPINION: only 13% of respondents were in favor of DTC-GT.

Mainous, 2013 (US) [102]	To examine academic family physicians' perception of and experiences with clinical genetic testing and DTC-GT.	1404 physicians most in clinical care.	45	Survey (quantitative)	AWARENESS: 54% of respondents felt that they were not knowledgeable about available GTs. ATTITUDE: They perceived utility for breast cancer (95%), Alzheimer's disease (30%), heart disease, or diabetes (25%). Individuals with greater self-perceived knowledge of GTs were more likely to feel that GTs would have a significant impact on their future practice (23%) than those with less knowledge (13%).
McGowan, 2014 (US) [97]	To assess how and why early clinical users of genomic risk assessment incorporate these tools in their clinical practices and how they interpret genomic information for their patients.	18 clinicians	23	Interview (qualitative)	OPINION: Many early-adopting clinical providers of SNP-based genomic testing were enthusiastic about the potential for these tests to enhance the personalized, preventive and wellness orientations of their clinical practices. EXPERIENCE: They largely did not have sufficient knowledge of genomics to independently help their patients manage the genetic risk information that commercial genomic analysis could provide.
Noss, 2014 (US) [103]	To investigate whether genetic counselling programs are incorporating instruction about the applications and techniques of predictive genomic testing (PGT).	114 genetic counselors who graduated between 2008 and 2011.	13	Survey (quantitative)	KNOWLEDGE: 68% of participants indicated that their training had incorporated PGT. A statistically significant increase was observed from 2008 to 2011 regarding incorporating information about DTC-GT. 63% of respondents indicated being taught how to discuss results.
Ohata, 2009 (Japan) [111]	To know the opinions of health care providers in Japan on new genetic testing services, such as DTC-GT	1145 general practitioners (GPs); 294 CGs	15% in the GPs group and 58% in CGs group	Survey (quantitative)	AWARENESS: 38% of GPs and 68% of CGs were aware of DTC-GT. EXPERIENCE: 0.5% of GPs and 1% of CGs performed DTC-GT. OPINION: GPS rated the benefit of DTC-GT more highly than CGs. CGs showed greater concern with regard to reliability of the results, provision of information/counselling and understanding of results. Awareness of DTC-GT enhanced GPs' positive opinion of it.
Ormond, 2011 (US) [104]	To assess knowledge, attitudes, and beliefs of students toward personal genomics and determine the impact of the course on this subject on knowledge, attitudes, and belief.	86 students enrolled in a human genetic course at Stanford University School of Medicine	48% completed the first survey; 36% the second survey.	Survey (quantitative)	OPINION: after the course students were less likely to believe that genotyping information would be useful to physicians (32% post course vs 63% pre course) and consumers (52% vs 84%). The greater concerns about DTC-GT included worries about reliability and utility of results. The majority of the students, both before and after course, agreed that tests needed interpretation (around 70%) and DTC companies had to provide a genetic counselling (71% pre- and 80% post-course).

Powell, 2012<br>(US) [105]	To assess primary care physicians' awareness, experience, opinions and preparedness regarding DTC-GT.	382 family physicians and internists members of the North Carolina Medical society.	16	Survey (quantitative)	AWARENESS: 39% were aware of and 15% felt prepared to answer questions about DTC-GT. EXPERIENCE: 19% of who were aware of DTC-GT had discussed DTC-GT results with their patients. OPINION: The majority of respondents (>80%) expressed concerns about misunderstanding of test results and increased anxiety in patients. Of respondents who were aware of DTC-GT 43% thought that testing was clinically useful.
Powell, 2012<br>(US) [106]	To assess the educational needs of primary care physicians about DTC-GT.	382 family physicians and internists members of the North Carolina Medical society.	16	Survey (quantitative)	OPINION: As above 85% felt unprepared to answer patient questions about DTC-GT and 74% of respondents reported wanting to learn about them. Physicians who felt either unprepared to answer patient questions or that DTC was clinically useful were more likely to be educated on this subject.
Ram, 2012<br>(New Zeeland) [112]	To explore the attitudes of GPs towards DTC-GT and elicit it their perceptions of the risks and benefits associated with DTC-GT.	113 registered GPs from a list provided by the New Zealand Medical Council	38	Survey (quantitative)	AWARENESS: 48% were aware of DTC-GT. OPINION: 72% of respondents who had not received training in genetics agreed that convenience was a benefit, compared to 38% of those with training ($p < .001$). Misunderstanding of results and inadequate provision of information were perceived to be the greatest risks associated (around 90% in both groups). The majority of respondents thought it was most appropriate to provide genetic counselling to a patient following a DTC-GT, though a genetic specialist (61%). Most of the respondents (79%) agreed that advertising of DTC-GT should be regulated in a similar way to DTC advertising of medicines. Clinical validity of tests (25%) and counselling (20%) were the more selected aspects the respondents thought to be regulated.
Salm, 2014<br>(US) [107]	To explore neurologists' and psychiatrists' knowledge, attitudes, and practices concerning GT	163 neurobiologists and 372 psychiatrists of the American Medical Association	7	online survey (quantitative)	EXPERIENCE: 74% of neurologists and 14% of psychiatrists had ordered a GT for a patient in the 6 months pre-survey; 15% of respondents had received inquiries about DTC-GT.<br>Most respondents reported deficits in their knowledge of genetics. 84% reported that they need more training in how to interpret test results.<br>OPINION: almost half of respondents (46%) thought that GT could cause psychological harm for their patients; 47% thought that legal protections against genetic discrimination are not adequate.

Weir, 2010 (CAN) [96]	To understand the knowledge and attitudes of Canadian health care professionals regarding nutritional genomics and nutrigenetic testing.	Four focus groups were held in two large Canadian cities including dietitians, naturopaths, nutritionists, physicians, and pharmacists.	NA	focus group discussions (qualitative)	OPINION: health care professionals had little knowledge about nutritional genomics and held contradictory attitudes towards genomics in general, and nutritional genomics in particular. They agreed that health care professionals acted as intermediates. They were also aware of their lack of competency to provide such services.

AUS: Australia; DTC-GT: direct to consumer - genetic test; CAN: Canada; NA: Not available; UK: United Kingdom; US: United States.

Table 4. List of articles on the content of websites offering direct-to-consumer genetic tests[a].

Reference	Sites analyzed, n	Provision of genetic counseling	Advice for physician's consumer supervision	Provision of recommendations associated with disease or health condition	Testing risks	Benefits of/reasons for testing	Provision of information on test limitations	Provision of information on clinical validity	Referral to laboratory certifications	Provision of privacy policy information
Arribas-Ayllon, 2011 [113]	3	-	-	-	-	A common strategy used by the companies is user empowerment	-	-	-	-
Borry, 2010 [118]	29	-	-	-	-	-	-	-	-	55%
Covolo, 2012 [124]	30	50% pre- and/or post-test	84%	-	30%	Empowerment of patient in 70% of cases	-	-	37%	-
Einsiedel, 2009 [128]	22	-	-	-	18%	Empowerment was consistently emphasized	-	38% provided some indication of evidence base	64%	-
Geransar, 2008 [123]	24	33%	67%	-	-	-	-	50%	-	-
Goddard, 2009 [115]	5	80% pre- and 20% post-test	60%	-	20%	40%	0%	0%	80%	80%
Gollust, 2003 [125]	14	21% by ge-tic counselor and 21% by physician	29%	7%	36%	-	-	-	-	-
Harris, 2013 [126]	20	30%	-	-	-	-	-	-	-	-
Hennen, 2010 [114]	38	26% (of which 60% pre-test - 80% post test	33% suggested the supervision of doctor 19% required doctor consultation	-	37% regarding - negative test results; 47% regarding positive test results	-	-	24%	63%	40%
Howard, 2011 [119]	13	31%	31%	-	-	-	-	-	-	23%
Kechagia, 2014 [120]	26	-	-	-	-	-	-	-	42%	Many of the websites analyzed

										provided limited information about Privacy Policy, consent procedures and use and storage of samples
Lachance, 2010 [121]	29	14% pre-test - 28% post test	21%	-	-	90%	55%	38%	45%	80%
Lewis, 2011 [122]	25	-	-	60%	16%	-	48%	40%	60% (of 20)	84%
Liu, 2008 [116]	46[b]	39% (4% pre-test)	-	15%	15%	The most popular emotional appeals were warmth and empowerment (61%)	-	54%	-	72%
Singleton, 2012 [127]	23	-	70%	74%	35%	96%	78%	-	-	-
Sterling, 2008 [117]	29[c]	0%	38%	53% (of 64)	31%	73% (of 64)	10%	17%	28%	72%

[a]All the percentages in the table represent the number of websites containing the specific information and were calculated on the basis of the total number of websites analyzed in the article.

[b]Of 46 websites, 20 allowed consumers to order directly to the company.

[c]29 organizations specifically offered or promoted at-home testing out of 64 organizations identified.
